# Addressing the factor associated with high rate of admission in a sample of patient with psychosis including the Changes Due to COVID-19 (Telepsychiatry)

**DOI:** 10.1192/j.eurpsy.2023.2309

**Published:** 2023-07-19

**Authors:** S. K. I. M. Aboelenien, K. Meshreky, H. Abdelkhalek, M. Karale

**Affiliations:** 1 CMHT, Essex Partnership university trust (EPUT), Rochford; 2 CMHT, Essex Partnership university trust (EPUT), Harlow; 3 CMHT, Essex Partnership university trust (EPUT), Wickford, United Kingdom

## Abstract

**Introduction:**

Patients with psychosis are particularly more prone to relapse, emergency presentation and subsequent hospital admissions (1). Hospitalization is not only a stressful experience to patients and carers, but also a financial burden to the National Health Service (NHS). Inpatient and community-based mental health services represent 47% of the annual healthcare costs for patients with SMI in the UK (2). The COVID pandemic had profound effects on health care services including the shift to remote consultations (Telepsychiatry) using video and telephone consultations in outpatient clinics. This shift to tele psychiatry was considered a novel challenge to both service users and service providers. We studied the impact of this change in the delivery of care on the number of admissions in patients suffering from psychoses.

**Objectives:**

We aimed at examining the factors associated with higher rates of hospital admissions and the effect of telepsychiatry (changes due to COVID) on the rate of hospital admissions in patients with a psychotic illness.

**Methods:**

We reviewed the care plans of forty patients with a diagnosis of a psychotic illness who were randomly selected from two specialist psychosis teams in Essex. We looked into a number of factors to identify their associations with the number of hospital admissions. Moreover, we compared patients who had changes in their care due to COVID-19 with those who received care as usual in terms of the number of admissions in the first year of the pandemic.

**Results:**

Patients who were under CTO or were receiving Clozapine had significantly higher total number of admissions compared to those who were not. The change in the type of care to telephone or video consultation during COVID-19 was associated with a lesser number of admissions during the first year of the pandemic.

Patients receiving clozapine have higher total number of admissions. This finding might be explained by the severity of illness in this patient group. It could also be linked to the re-initiation policy within EPUT that mandates inpatient initiation and re-initiation of clozapine even in the absence of relapse signs. The shift from classic face-to-face to telephone and video consultation has its well-known shortcomings. However, it does appear to be a suitable alternative to a group of patients that it was associated with lower rates of hospital admission during the height of the pandemic.

**Image:**

**Figure:**
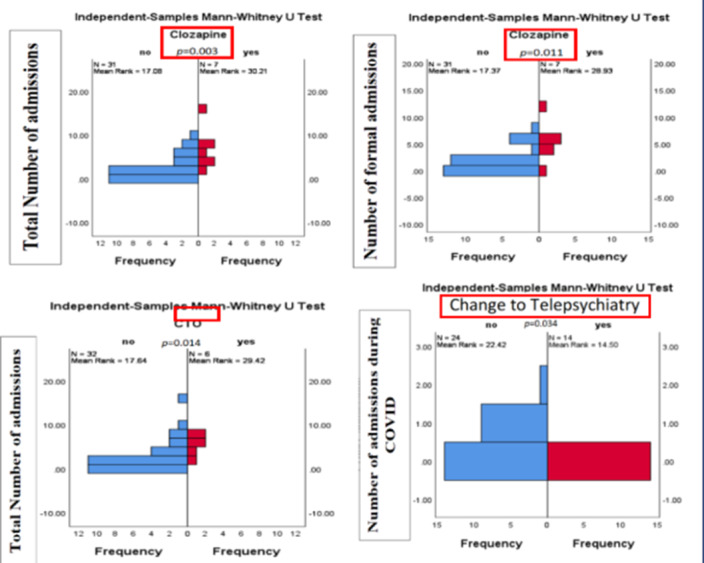

**Conclusions:**

To conclude, the factors that were associated with a higher number of admissions in patients with psychosis were under CTO or receiving treatment with Clozapine. Preliminary evidence showed that telepsychiatry is a suitable alternative to standard care during the COVID-19 pandemic.

**Disclosure of Interest:**

None Declared

